# Equilibrium landscape of ingress/egress channels and gating residues of the Cytochrome P450 3A4

**DOI:** 10.1371/journal.pone.0298424

**Published:** 2024-03-18

**Authors:** Edward Michael Ackad, Laurence Biggers, Mary Meister, Maria Kontoyianni

**Affiliations:** 1 Department of Physics, Southern Illinois University Edwardsville, Edwardsville, IL, United States of America; 2 Department of Internal Medicine at UT Southwestern Medical Center, Dallas, TX, United States of America; 3 University of Illinois College of Medicine, Chicago, IL, United States of America; 4 Department of Pharmaceutical Sciences, Southern Illinois University Edwardsville, Edwardsville, IL, United States of America; Universidade Nova de Lisboa Instituto de Tecnologia Quimica e Biologica, PORTUGAL

## Abstract

The Cytochrome P450 (CYP) enzymes metabolize a variety of drugs, which may potentially lead to toxicity or reduced efficacy when drugs are co-administered. These drug-drug interactions are often manifested by CYP3A4, the most prevalent of all CYP isozymes. We carried out multiple MD simulations employing CAVER to quantify the channels, and Hidden Markov Models (HMM) to characterize the behavior of the gating residues. We discuss channel properties, bottleneck residues with respect to their likelihood to deem the respective channel ingress or egress, gating residues regarding their open or closed states, and channel location relative to the membrane. Channels do not display coordinated motion and randomly transition between different conformations. Gateway residues also behave in a random fashion. Our findings shed light on the equilibrium behavior of the gating residues and channels in the apo state.

## 1. Introduction

The Cytochrome P450 (CYP) enzymes in the human liver metabolize a wide variety of drugs, which can lead to toxicity or reduced efficacy when drugs are co-administered. These drug-drug interactions are often manifested by CYP3A4 which is the most prevalent of all CYP isozymes, and responsible for the biotransformation of more than one-third of currently marketed therapeutics. Regarding CYP3A4, there is evidence that: (1) ligands bind to sites other than the active site; (2) more than one ligand can bind to it; (3) the isozyme is plastic and exists in multiple conformations in a dynamic equilibrium, and (4) a membrane-bound structure is more optimal to study physiologically-relevant access channels.

In reference to point (1) above, most crystal structures have the ligand bound in the active site, but only two complexes show the respective substrates in productive modes [1, 2]. Nonetheless, the substrate progesterone was resolved in the periphery 17 Å from the active site, with no ligand close to the heme [3]. Atypical CYP3A4 kinetics was observed with nifedipine and testosterone, where nifedipine inhibited the 6β-hydroxylation of testosterone, but testosterone did not inhibit the oxidation of nifedipine [4]. The explanation for these results is that nifedipine can bind to multiple sites of CYP3A4, while testosterone is fixed to a certain domain within the site. Hosea and colleagues [5] performed binding studies with several peptides, and also steady-state analysis with several substrates, and concluded that CYP3A4 has at least two, and probably three binding sites with somewhat distinct features. With respect to inhibitors, they are in the active site in most crystal structures. However, the CYP3A4-ketoconazole complex shows two molecules stacked in an antiparallel orientation against one another [6]. Davydov et al., [7] employed fluorescence resonance energy transfer and identified for the first time a peripheral site outside of the active site, the location of which is similar to the position of progesterone in its crystal complex with CYP3A4 [3]. These results provide evidence that ligands can bind to sites which are not close to heme in CYP3A4.

Regarding multiple substrate binding, it is believed that one molecule binds at a peripheral site, and subsequently the substrate either moves toward the heme (active site) or a second ligand enters the active site and interacts with the heme [8, 9]. An alternate binding model has been proposed where both substrates compete for one (low-affinity) site, and once that site is occupied the second substrate binds to an allosteric site [10]. Inhibitor binding has also been postulated to follow a three-step sequential or a three-step two-ligand sequential binding model [11]. Finally, Polic et al. [12] experimentally demonstrated that CYP3A4 mutants, covalently modified with various small molecules such as progesterone, exhibited enhanced kinetic stability in testosterone and 7-benzyloxy-(4-trifluoromethyl)coumarin oxidation assays. Therefore, their work supported the hypothesis that a postulated allosteric site of CYP3A4 induces functional cooperativity upon substrate binding. Together these studies show that multiple ligands can bind to CYP3A4.

CYP3A4 is malleable and exists in many conformational states with a dynamic equilibrium among them. Studies have shown that the metabolism of benzo[a]pyrene was enhanced by 7,8-benzoflavone, [13, 14] because benzo[a]pyrene bound to a different conformer of CYP3A4 in the presence of the flavonoid [15]. Similarly, metabolism of diclofenac and warfarin is enhanced in the presence of quinidine, while 7, 8-benzoflavone inhibits the metabolism of both, diclofenac and warfarin [16–18]. The differences between 7, 8-benzoflavone and quinidine towards CYP simulation were attributed to changes in enzyme conformations due to effectors. Thus, the equilibrium between CYP3A4 conformations responsible for the metabolism of diclofenac or warfarin is perturbed. Similarly, nifedipine and quinidine bind to different conformations of CYP3A4 [19]. These findings provide evidence of multiple pre-existing conformations of CYP3A4.

With respect to advantages of membrane presence, a study comparing the crystal structures of membrane-bound and soluble CYPs revealed that the A-propionate side chain of the heme is shifted towards the proximal side of the heme plane, contrary to soluble structures where the A-propionate points to the distal site [20]. This results in an increase of the volume of the active site in membrane-bound CYPs. Furthermore, while the F-G helical bundle is thought to be the main opening for binding and product release, study of the crystal membrane-bound CYPs shows they share a longer F-G loop and extra F′ and G′ helices, involved in interactions with the membrane bilayer; this shift allows for the direct access of lipophilic substrates from the membrane interior. Similar conclusions were reached by another investigation of several membrane-anchored CYPs, which showed that the catalytic domains are partially immersed in the lipid bilayer, while the N-terminal and the F’-G’ loop are deeply immersed [21]. Finally, a comparison of membrane-bound and soluble CYP2C9 showed that the membrane stabilized protein conformations conducive to opening access channels [22]. These findings provide evidence that a membrane-bound system is preferred over a soluble CYP.

We thus set out to explore (1) the dynamic landscape of CYP3A4 and (2) the extent to which the gating residues are open or closed as the isozyme is undergoing conformational transitions. Studies focusing on the mechanisms associated with egress from CYP3A4’s active site are scarce. A few studies, which will be discussed in more detail herein, have employed steered molecular dynamics (MD) and Hamiltonian replica exchange MD to investigate metabolic product routes accommodating their exit from the enzyme. Fishelovitch et al. [23] reported on preferred egress product pathways and gating mechanisms using steered molecular dynamics. Pulling the products temazepam and testosterone-6βOH out of the P450 3A4 enzyme, they located six egress pathways with different exit preferences for the two products; they also found more than just one access/exit channel in CYP3A4. Their notation for the six channels (2a, 2b, 2c, 2e, 3 and S) followed that of Cojocarus et al’s work on CYP2C9 [22]. The solvent channel S manifested the largest opening for both products, and thought to be a putative substrate channel. They also reported that channel properties depended upon the ligand. Paloncýová et al. [24] investigated the passage of a ligand to the active site of CYP3A4 using a combination of bioinformatics tools and bias-exchange metadynamics. Even though they found seven channels using MOLE2.0 [25], they only explored three since they were the only ones that stayed open for more than 1ns during the initial 100 ns MD trajectory. Their study focused on the changes these channels underwent during egress of 1,3,7-trimethyluric acid. Further, in regards to approaches for channel identification, strategies involve (i) grid-based methods, which project macromolecules onto a 3D grid, process the void volumetric pixels and then connect them into channels. Examples include POCKET [26], LIGSITE [27, 28], HOLLOW [29] and CHUNNEL [30]; (ii) optimization methods, which split the macromolecule and subsequently perform optimization to identify the largest spheres. Examples are HOLE [31] and PoreWalker [32]; (iii) methods employing Voronoi diagrams of the centers of fixed-size balls which are used to represent the macromolecule, followed by an approximation of the medial axis to search for the shortest path from a starting point to the surface. Representative software tools using this methodology are MolAxis [33, 34], CAVER 3.0 [35] and CCCPP [36]; (iv) sphere-filling methods, in which a cluster of spheres is considered a pocket, are implemented in PASS [37] and SURFNET [38].

We present herein our findings from multiple MD simulations employing CAVER to quantify the channels. Once metabolites (products) are formed, product egress presupposes they exit from the catalytic, near heme, site through channels to either the membrane or the cytosolic environment. A cluster of mostly phenylalanine residues found by Fishelovitch et al. [23] control the access of the products to the channels. By understanding the equilibrium behavior of these so-called gating residues for the apo state, we shed light on parameters deterministic of ligands favoring one channel over another. We apply an HMM [39] methodology to characterize the behavior of the gating residues for the cytochrome P450 3A4. HMM have been used in genome studies, [40] protein folding, [41] identification of meta-stable states, [42] protein functional mechanisms, [43] and the detection of binding sites [40]. Our HMM model and analyses allow for quantification of the gating residues’ behavior and assessment of the most likely metabolite egress channels. Moreover, dissemination of this apo model will provide a foundation for comparative studies against bound (holo) complexes with ligands in the periphery.

## 2. Methods

### 2.1. Protein preparation and MD

The starting structure, consisting of the equilibrated 1-palmitoyl-2-oleoyl-sn-glycero-3-phosphocholine (POPC) membrane-bound CYP3A4, was generously shared with us by Professor Sligar’s group [44]. They started with the apo crystal CYP3A4 (PDB 1TQN) structure, then equilibrated and simulated it without restraints for 100ns. We subsequently rehydrated the structure, and neutralized it using VMD tools resulting in a system 120x120x145 Å in size and with approximately 140,000 atoms. The system was then minimized using 50,000 steps with CHARMM36 force field, where the heme parameters were obtained from CHARMM-GUI. Non-bonded interactions used a switching distance of 10 Å with cutoff at 12 Å and a pair-distance of 16 Å. Afterwards, all non-water atoms and ions were fixed, and MD simulations were performed at 310 K for 100 ps using a 1 fs timestep. All non-water atoms and ions were then constrained by a harmonic potential of 1 kcal/mole/Å^2^, and run for an additional 100 ps at 310 K with a 1 fs timestep. Next, we restrained the protein backbone and heads of the POPC lipids with a 1 kcal/mole/Å^2^ harmonic restraint, while the rest of the non-water atoms or ions were only restrained by a 0.7 kcal/mole/Å^2^ harmonic constraint. The values of the constraints were gradually decreased to: 0.5, 0.2, 0.1, and then 0 kcal/mole/Å^2^, with the protein backbone and lipid heads remaining more restrained than the rest of the protein and lipid tails. Each of these equilibrium steps were run for 100 ps at 310 K with a 1 fs timestep for each set of constraints. All simulations were performed as an NVT ensemble using Langevin dynamics with a piston period of 50 fs, a decay time of 25 ps at a target pressure of 1.01325 bar, and target temperature of 310 K. Production runs were done at NPT using typical Langevin dynamics, without restraints and with a 2 fs timestep and NAMD’s [45] rigid bonds (restricting the length of hydrogen atoms). Sixteen independent trajectories were run amounting to a total of 4400 ns.

### 2.2. Channel determination

The channels were determined using CAVER Analysts 2.0 Beta 2. [46] The MD trajectories were stripped of everything except the protein and heme, and combined into a single file using catDCD 5.2. RMSDVisualizer in VMD was employed to align the protein via the Cα’s only. The aligned trajectory was used for analysis in CAVER Analyst with a total of 3313 frames. The reduced number of frames was to accommodate the memory limited and was achieved by only using every 100^th^ frame (dcd skip of 100).

Starting location for the channels was found using CAVER’s [35] cavity computation with a probe of 2.6 Å radius on the first frame. The largest cavity was located adjacent to the heme, and used to create the starting point for channel computations. A large shell radius of 5 Å was selected to ensure the “outside” region did not enter any of the access channels. The shell depth was set to 4 Å to disallow channel splitting near the surface of the protein, while minimum probe radius was 1.0 Å to ensure that the most dominant channels were identified. It is notable that reducing this value results in an increased number of frames in which a channel is observed. The number of approximation balls was set to 20 in order to best represent sidechains. The trajectory was sampled every 2ns due to memory constraints.

CAVER [35] reports multiple measurements about each channel under investigation. These include the number of snapshots the channel is found in, the average length of the channel (calculated along the channel’s axis), the average curvature of the channel (length divided by the straight-line distance to the channel’s end point), and the maximum and average bottleneck radii. In addition, the custom metrics of throughput and priority are calculated. Throughput is a measure of how easily (from 0 to 1, with 1 being the easiest) a ligand is able to traverse the channel; ideally the channel should be as short and as wide as possible. The priority of a channel is the average throughput calculated from all snapshots with 0 (worse throughput) corresponding to snapshots where the channel was not detected, and it thus provides a measure of how favorable the channel is or likely to serve as egress as it relates to its plausible flux.

### 2.3. Bottleneck analysis

The results from CAVER’s bottleneck analysis were used to determine the frequency of each residue’s participation in the bottleneck cluster. The set of residues that comprised the bottleneck was noted at each frame and for each channel. All possible three-residue combinations, termed “trios” from this point onward, and their occurrence frequency were then found. Neighboring residues (three residues away or less) were not counted to avoid trivial associations. The ten most frequently occurring residue clusters were then normalized to the number of total frames in which the channel occurs, using $f_{i}=\frac{c_{i}}{N}$, where *f*_*i*_ is the frequency of occurrence of the *i*-th trio, *c*_*i*_ is the number of frames the *i*-th trio was found to be a bottleneck, and *N* is the total number of frames in which that channel was found. The distance from the location of the bottleneck, as recorded by CAVER, to the starting point was calculated for each residue trio’s first occurrence, and used to set the shade of blue in respective bar charts. The distance value was normalized to the furthest cluster found. Additionally, the radii of each frame’s bottleneck were recorded for every channel, and the results were binned and sorted by the residue trio for all possible combinations of residues in that bottleneck cluster. The 15 most frequent residue trios were identified by determining the total number of radii found.

### 2.4. Channel exit calculations

The membrane was constructed in the x-y plane of the simulation box. Thus, the z-value of a coordinate represents the distance perpendicular to the plane of the membrane. Only the monolayer in contact with the embedded F’ and G’ helices was used for the calculations described in this section. A location (three-space coordinate) must be assigned to every channel (for each frame) in order to determine the channel’s exit with respect to the membrane. This was done by finding the midpoint between two alpha carbon atoms (Cα) of each exit’s residues, listed in Table 1.

**Table 1:** Exit residues used to calculate the location of the channels’ exits with respect to the membrane.

Channel Name	Gating Residues
2a	51–216
2b	106–227
2c	113–247
2e	107–122
3	219–233
S	212–484

The height of every channel’s exit above different moieties constituting the membrane was subsequently calculated. Each plane was constructed by averaging the z-values of the monolayer atoms (nitrogen for the head-group layer, 〈*z*_*H*_〉, carbon for the tail layer, 〈*z*_*T*_〉) into which the F’ and G’ helices were embedded. For instance, the z-value (height in the simulation box) of the nitrogen for each frame represents the height of this particular atom’s plane, 〈*z*_*N*_〉. The z-value of the channel, *z*_*c*_, was then subtracted from this number to calculate the height *above* the nitrogen plane: *h*_*i*_ = (*z*_*c*_)_*i*_−〈*z*_*N*_〉_*i*_ for the *i*th frame. For the tail, carbon atom 211 (tail-carbon) α to the double bond was used. Similarly, the radial distance calculations used the same exit location. The minimum radial distance to the closest POPC nitrogen was then found by measuring all distances to POPC nitrogen atoms and subsequently calculating the minima. The same approach was used for the nitrogen and tail-carbon atoms.

### 2.5. Hidden Markov state model building

Dimensionality reduction was achieved employing the previously identified [23, 39] six pairs of gating residues (Table 2) or each channel using a contact featurizer in MSMBuilder. The featurizer used the closest atoms’ method to determine the contact distance and included hydrogens. Time-lagged independent component analysis (TICA) was then employed with a lag time of 20 ps to transform the six distance coordinates using PyEMMA’s [47] tica function. TICA is a linear transformation of the given coordinates to a smaller set of coordinates that maximizes the autocorrelation, thus creating a subspace which encompasses as much of the information as possible in as few coordinates as possible. This is done by solving the generalized eigenvalue problem:

**Table 2:** Exit channels in CYP3A4 and respective gating residues [50].

Channel Name	Gating Residues
2a	F57-F215
2b	F108-F220
2c	F108-F241
2e	F108-I120
3	F213-F241
S	R212-L482


C(τ)U=C(0)UΛ,

where, *τ* is the lag time, ***C***(*τ*) is the covariance matrix between the input data at *τ* = 0 and *τ*, *U* is the generalized eigenvector matrix with the independent components as the columns, and **Λ** is the eigenvalues matrix (which is diagonal). Projection of the previous coordinates to the TICA coordinates is then accomplished by multiplying the transpose of the original coordinate vector with the eigenvector matrix ***U***. The new six TICA coordinates maximized the autocorrelation for the channels gating residues minimum distance (see SI for the channel to TICA axis correlation matrix).

The following parameters need to be selected for an HMM model: (1) The number of clusters to be generated from the featured data, (2) the lag to be used, and (3) the number of HMM states. Multiple measures were considered in order to distinguish among the resultant models in an effort to construct the most stable one. An obvious metric at first was the root-mean-squared-deviation (RMSD) of some representative snapshots.

$$R_{j}=\sum_{i=1}^{50}RMSD_{ij}$$

where *j* is the gating residue pair and *i* is summed over 50 representative frames obtained using the sample_by_observation_probabilities function in PyEMMA. This measure carries an inherent bias that HMM models with more states will give larger RMSDs. Thus, the measure was normalized to the number of HMM states. Results for each channel show that the increasing number of HMM states only slightly lowers the total RMSD of the channel (see results in SI). This is consistent with all tested cluster sizes (50, 64, 100, 200) and lagtimes (20 ps, 100 ps, 200 ps).

The metric used to choose the three parameters was the fractional uncertainty between the left and right eigenvectors of the stationary distributions (Eq 1)

$$f=\frac{\left(\sum_{i=0}^{N}\frac{\left|\mu_{i}-\nu_{i}\right|}{\mu_{i}}\right)}{N}$$ (1)

where *N* is the number of states in the HMM mode, *μ*_*i*_ is the *i*th component of the left eigenvector, and *v*_*i*_ is the *i*th component right eigenvector of the transition matrix with eigenvalue *λ* = 1. The sum should be zero since the transition matrix is a stochastic matrix. The fractional uncertainty is minimized using 64 clusters, a lag time of 20 ps, and a 10-state HMM. A plot of *f* for HMM models with six to twenty states is included in the SI for each of the 1400 models (100-models for each number of HMM states) showing a clear minimum with a 10-state model. Of the 100 10-state HMM-models, we picked the one with the smallest *f* for the analysis of conformations, but used all 100 for the transition calculation.

The calculations work by first defining the size of the boundary between open and closed conformations ranging from 4.3 Å to 5.6 Å in increments of 0.025 Å. For each of the 100 HMM models, 1000 representative observations were picked using the “sample_by_observation_probabilities” function in PyEMMA. Each channel had its average gating distance measured, and was subsequently labeled *open* or *closed* based on the boundary. The total flux was then measured from all closed states to open states, using the transition path theory function for HMM. Mean and standard deviations for the 100 HMM models are recorded and used in the analysis. The Chapman-Kolmogorov test is another way to verify the behavior of a model; however, it is limited in that it can be useful for a few lag-time multiples given we are modeling the fast, rather than the slowest, process. Therefore, results are insufficient to optimize the parameters.

## 3. Results and discussion

### 3.1. Channels

#### 3.1.1 Properties

**Fig 1:**
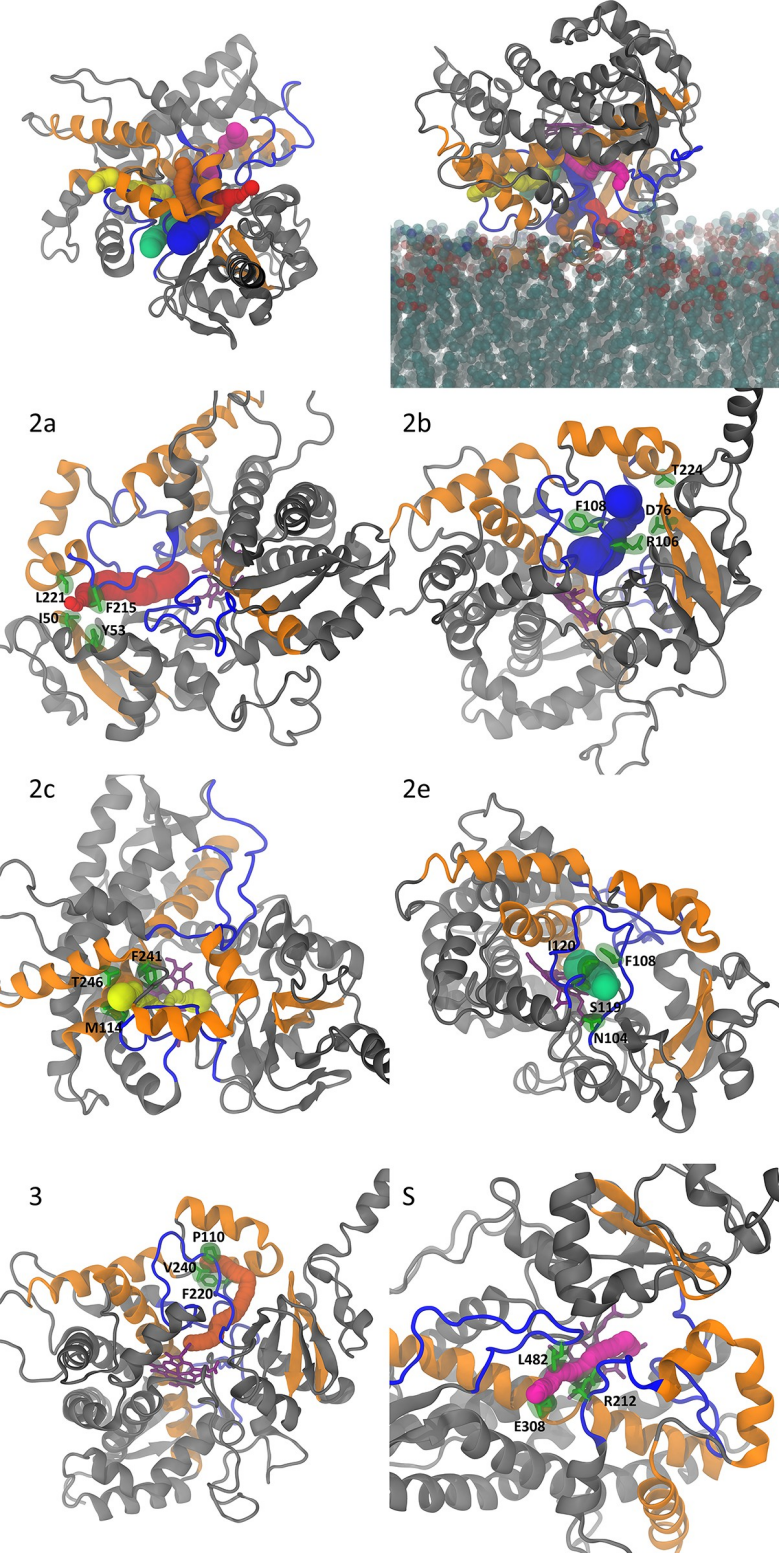
All six channels are depicted (2a red, 2b blue, 2c yellow, 2e lime green, 3 orange, and S magenta), without the membrane (top left) and with the POPC membrane (top right). Heme is in sticks and dark purple. The most frequent bottleneck residues are labeled and depicted as sticks with their van der Waals surface.

At the outset and in order to characterize the apo state’s equilibrium behavior of CYP 3A4, we set out to investigate the access channels. We employ the nomenclature presented in Table 2, since we detect the same major channels as previously reported [23]. These channels, along with their most frequent bottleneck residues, are given in Table 3 and depicted in Fig 1.

**Table 3:** Parameters for identified channels using CAVER.

**Channel**	Snps^a^	BR^b^	**SD**	**MaxBR**	Length^c^	**SD**	Curv.^d^	**SD**	**Priority**	Thrp^e^	**SD**
**2b**	3129	1.77	0.39	3.13	21.52	3.38	1.44	0.20	0.64	0.67	0.09
**2a**	2329	1.48	0.38	3.01	25.11	3.72	1.28	0.12	0.40	0.57	0.11
**S**	1855	1.40	0.32	2.80	21.86	5.36	1.38	0.20	0.31	0.56	0.12
**2e**	1603	1.35	0.23	2.18	15.75	3.34	1.27	0.18	0.30	0.62	0.08
**3**	527	1.44	0.39	2.47	27.31	5.49	1.65	0.31	0.09	0.54	0.12
**2c**	222	1.16	0.17	1.99	26.19	4.84	1.36	0.22	0.03	0.42	0.11

^a^Number of snapshots out of 3313.

^b^Average bottleneck radius.

^c^Average length in angstroms.

^d^Average curvature.

^e^Average throughput.

The most prevalent channel is 2b (3129 snapshots out of a total of all 3313 frames or 94%), which also displays the highest priority (0.63709), even though it has a significant overall curvature and is not the shortest channel. As can be seen, 2b was 24% more prevalent than the second most often observed channel, the 2a. Channel 2b also has the largest average bottleneck radius, almost 18% larger than that of 2a. The relatively small standard deviation of the throughput measure, with a fractional deviation of only 13%, and the prevalence throughout all simulations, strongly suggest that 2b is wide and open. These findings are in accord with Benkaidali et al., [36] who used the CCCPP server on 24 crystal structures and identified three conformations. The 2e is the shortest (15.75 Å in length) and the straightest channel. It is minimally open, with a bottleneck radius of 1.35 Å in roughly half of the snapshots in which it is detected. However, it has the second highest average throughput and the second smallest bottleneck radius. These findings together suggest that when the channel is open, a large fraction of small ligands, given the size of the radius, may traverse through the channel with minimal hindrance. Because the *maximum* bottleneck radius is also small, the second smallest indeed, it seems that the maximum distance of a set of residues (i.e., bottleneck) would be the determining and also limiting factor with respect to throughput. Fishelovitch et al. [23] reported that of all exit channels, 2e required the lowest and the second lowest amount of steered MD work invested to pull temazepam or testosterone-6βOH out, respectively. Even though our investigation focuses on an apo membrane-anchored CYP3A4 with extensive MD simulations, as opposed to their complexed CYP3A4 with a shorter steered MD approach, our findings agree with those of the aforementioned investigators. Thus, we suggest drugs prefer linear and short channels over long and wide ones. Channel 2c has a bottleneck radius larger than 1 Å in less than one tenth of the snapshots, while also displaying the smallest average bottleneck radius among all channels. It is the second longest channel with the lowest average throughput, whereas its largest bottleneck radius is the smallest among six channels. Consequently, it would seem that 2c is less easy to traverse relative to other channels. Our data are seemingly in agreement with results by Fishelovitch et al. [23] in that they found 2c requires the most work along the testosterone-6βOH trajectory for its exit. Further, these investigators reported that 2c is the second highest in steered MD work needed for temazepam’s exit from the catalytic site. Notably, the channels are dynamic and the property measurements reported herein change by more than 9%. The extent of the percent change is in accord with the reported plasticity of CYP3A4, and supports the notion of multi-step ligand binding[1, 8, 11] and existence of multiple conformational states [48, 49].

In summary, the size of ligands moving away from the heme toward the membrane or cytosol is seemingly correlated with channel throughput and curvature. Specifically, 2b displays the highest throughput, which could be attributed to or correlated with its large bottleneck radius. However, given that it also has the second largest curvature, we hypothesize large but flexible drugs preferentially exit through this channel. In contrast, channel 2c is the least likely to be traversed by ligands or metabolites exiting the catalytic site given its lowest throughput among all channels. This observation is also substantiated by 2c’s smallest average bottleneck and maximum bottleneck radii. Channel 2e is the likely exit channel for small and conformationally compact structures due to its relatively high throughput and smallest average bottleneck radius and curvature. Finally, channel 2a is an exit pathway for medium-size, flexible molecular structures. These results elucidate the behavior of the access channels in the apo state of membrane-bound CYP3A4. Notably, the highest throughput channels open up toward the membrane, as will be discussed in following sections.

#### 3.1.2 Bottleneck residues

**Fig 2:**
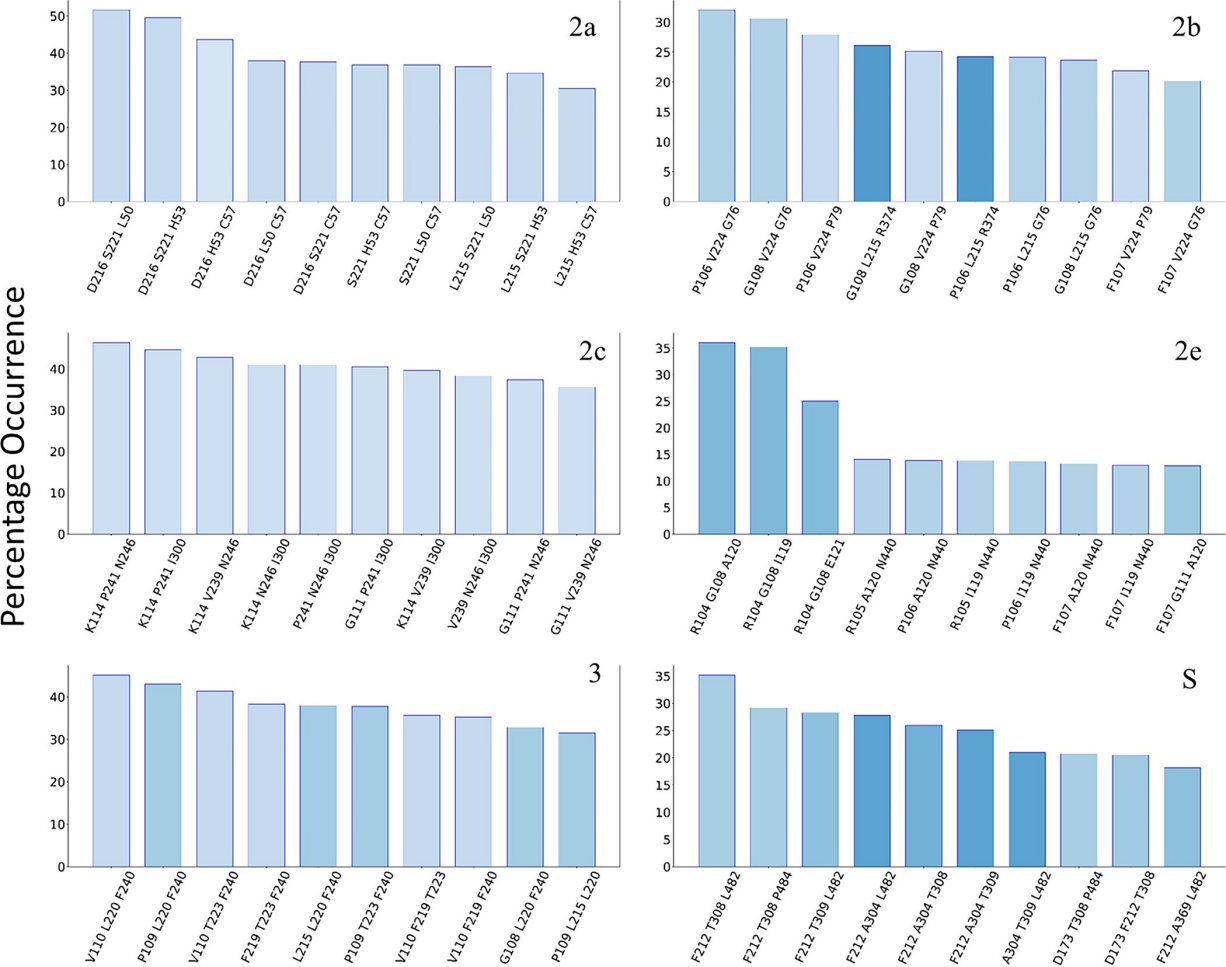
Percentage occurrence of the most frequent bottleneck residue trios. Shade of blue represents the distance from the heme, with darker being closer to the heme.

Fig 1 shows the channels and corresponding bottleneck residues, whereas Fig 2 displays the percent occurrence of the top ten most observed residue trios. We should emphasize that our focus is on three residues, rather than four, since the frequency of each three-residue cluster observed is significant (greater than 10%). The darker the color of the bar, the closer that particular group of bottleneck residues is to the catalytic (heme) site, for color represents the radial distance from the heme. It can be seen that the majority of residues cluster far from the catalytic site (lighter blue). Among all residue trios for channel 2a, the one consisting of F216, L221, and I50 is the bottleneck trio more than 50% of the time. Other frequently observed clusters include neighbors of the aforementioned residues and with all being around the exit. Because these residues are closer to the membrane, our findings are reinforcing to an extent earlier reports [24] that a significant free energy barrier separates the open from closed states. These investigators found that the free energy barrier driving 1,3,7-trimethylbutyric acid from the active site through the 2af channel (which corresponds to our 2a channel) was the largest at the exit; it also resulted in opening the channel’s radius by approximately 1.6 Å. Similarly, all bottleneck residues for 2c are found away from the heme, while inner residues account for less than 15% occurrence (see SI), even though this is the second longest channel. In contrast, bottleneck residues for 2e are mostly residues N104, F108, S119, I120, and A121, which are in the middle of the channel. These are the most dominant and likely to cause a bottleneck in combinations of three. For the remaining channels (2b, 3, S), the bottleneck residues are in various positions with no discernable pattern. In summary, 2a and 2c display bottleneck residues far from the heme, which inadvertently may be instrumental in closing a channel and preventing drug ingress.

#### 3.1.3 Bottleneck radii

**Fig 3:**
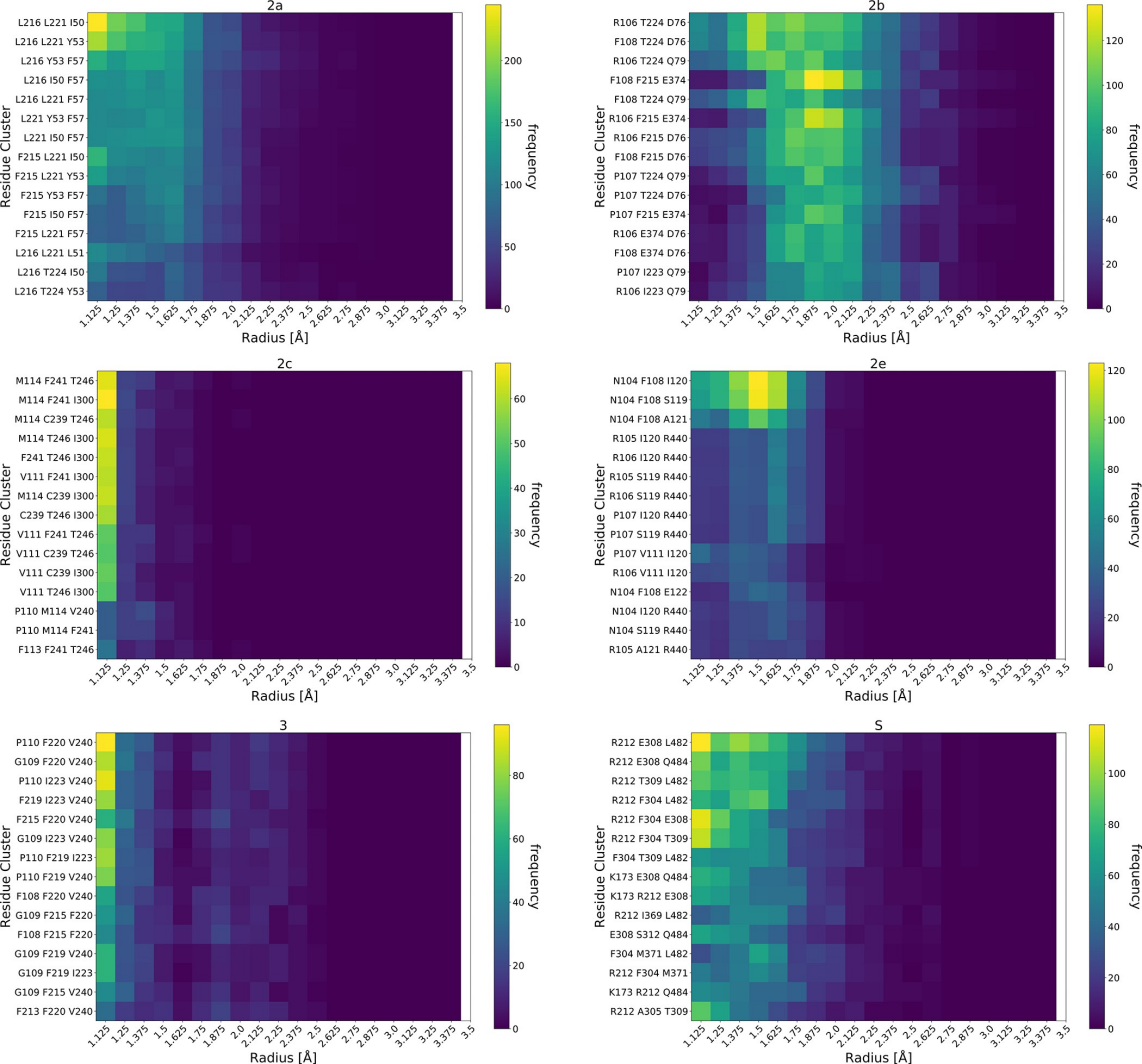
Heatmaps of the most frequent residue trios’ bottleneck radius distribution for each channel.

Fig 3 shows the radii distributions of the bottleneck residues plotted as heatmaps for each channel, with the most frequent residue trios at the top of each heatmap. The bottleneck residues in the 2e channel are N104, F108, and I120 and peak at a radius of 1.5 Å. The distribution is wide spanning from 1 Å to 1.9 Å. The bottleneck distributions in the 2c channel strongly peak just above 1 Å, which was the cutoff radius used. Further, the distributions in this channel display a clear decrease in frequency as the bottleneck radius increases. Channel 3 behaves in a similar fashion to channel 2c (main peak at 1 Å) with the exception of a secondary, smaller peak around 1.875 Å (notice the minimum at 1.625 Å) which is absent in 2c. The side-chains of Phe and Val of channel 3 have rotamers which can face away from the channel, allowing for larger distributions of radii, and thus accounting for channel 3’s second peak. In channel 2e, the bottleneck radii distribution for the top three trios is around 1.5 Å. Even the less frequent residue combinations peak at around 1.5 Å, most around 1.625 Å. An exception occurs when the bottleneck is at the boundary to the membrane or cytosol (Fig 2); these trios (including R106, R107, V111, I120) have distributions centering around 1.375 Å. Therefore, 2e will have its bottleneck midway through the channel with a fairly large radius of around 1.5 Å, but this can be replaced by the channel exit closing and becoming the bottleneck. The distributions in 2a are centered near 1 Å, but stretching to around 1.5 Å. The S channel has nearly the same distribution. Finally, 2b’s bottleneck radii distribution peaks at around 1.75 Å and is consistent for the 15 most frequent trios, despite their different locations throughout the channel. Thus, the 2b channel is consistently wide, regardless of where the bottleneck occurs in the channel.

In summary, 2a, 2c, and 3 channels have bottleneck residues the farthest away from the heme. However, in 2a the bottleneck radii extend more than the other two channels, in which the trios are more contained within a narrow radius. These findings are similar to our data discussed in section 3.1.2 regarding channels 2a and 2c. It would appear that bottleneck residues closer to the solvent or membrane are characteristic of channels which allow a metabolite to exit the enzyme. In contrast, if the bottleneck residues are further down the channel/path or even closer to the catalytic site, they would hinder the exit of the metabolic product, whereas they might be amenable for passage of a ligand entering the enzyme. The latter can be justified by the malleability of those passages, so that once a drug enters a channel, even if the bottleneck residues prohibit it from moving further, it will continue its path towards the heme once the radii widen, thus allowing ingress.

### 3.2. Location of channel exits with respect to the membrane

**Fig 4:**
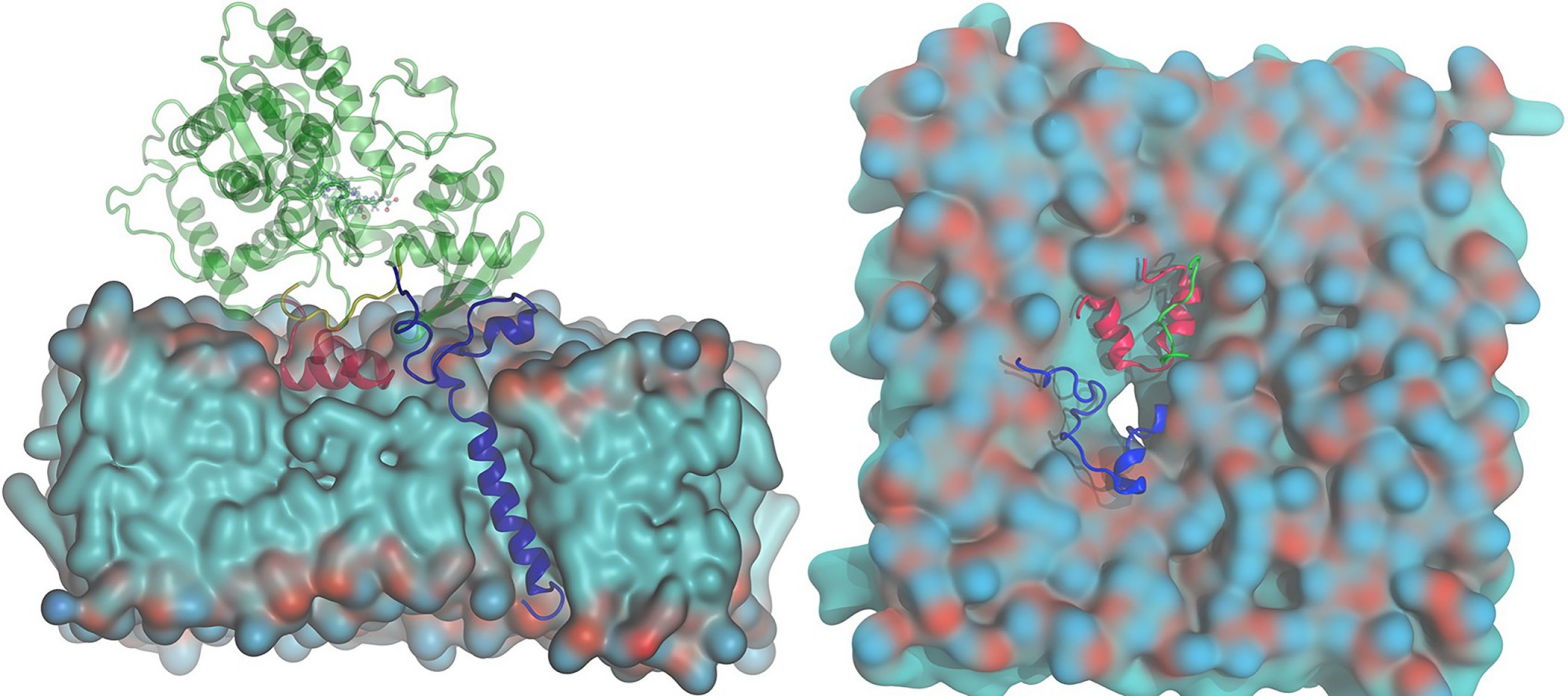
**Illustration of key structures (shown as cartoons) with the POPC membrane as a surface.** Blue cartoon depicts the end of the transmembrane helix, A helix, and β1 sheet. The F’ (left) and G’ (right) helices are in red, while the BC loop is shown in green and hovers above the G’ helix. The membrane’s red and dark green colors reflect the locations of phosophorus and nitrogen, respectively. Light green surface represents the tail of the POPC lipid.

The conformation CYP3A4 adopts relative to POPC bilayer is depicted in Fig 4. POPC is shown as a cross-section of the protein in the bilayer (left panel) with the F’ and G’ helices (red) and the β1 sheet (blue loop) forming a bowl-like indentation as they are embedded into the membrane at a depth just below the phosphate groups. The displaced head groups are pushed to the side, while the tails form the bottom of the bowl. The right panel illustrates the direct contact of the structure with the membrane and the BC loop (green). The N-terminus transmembrane anchor is blue, followed by the A helix and β1, while the BC loop (green) is above the G’ helix. Channel 3, located between the F’ and G’ helices, is the channel exiting most deeply into the membrane. The 2a channel, between the F’ and β1 sheet, is also deeply embedded into the membrane. The largest channel 2b has its exit between the G’ helix and BC loop, and thus opens up to the head group of the membrane.

**Fig 5:**
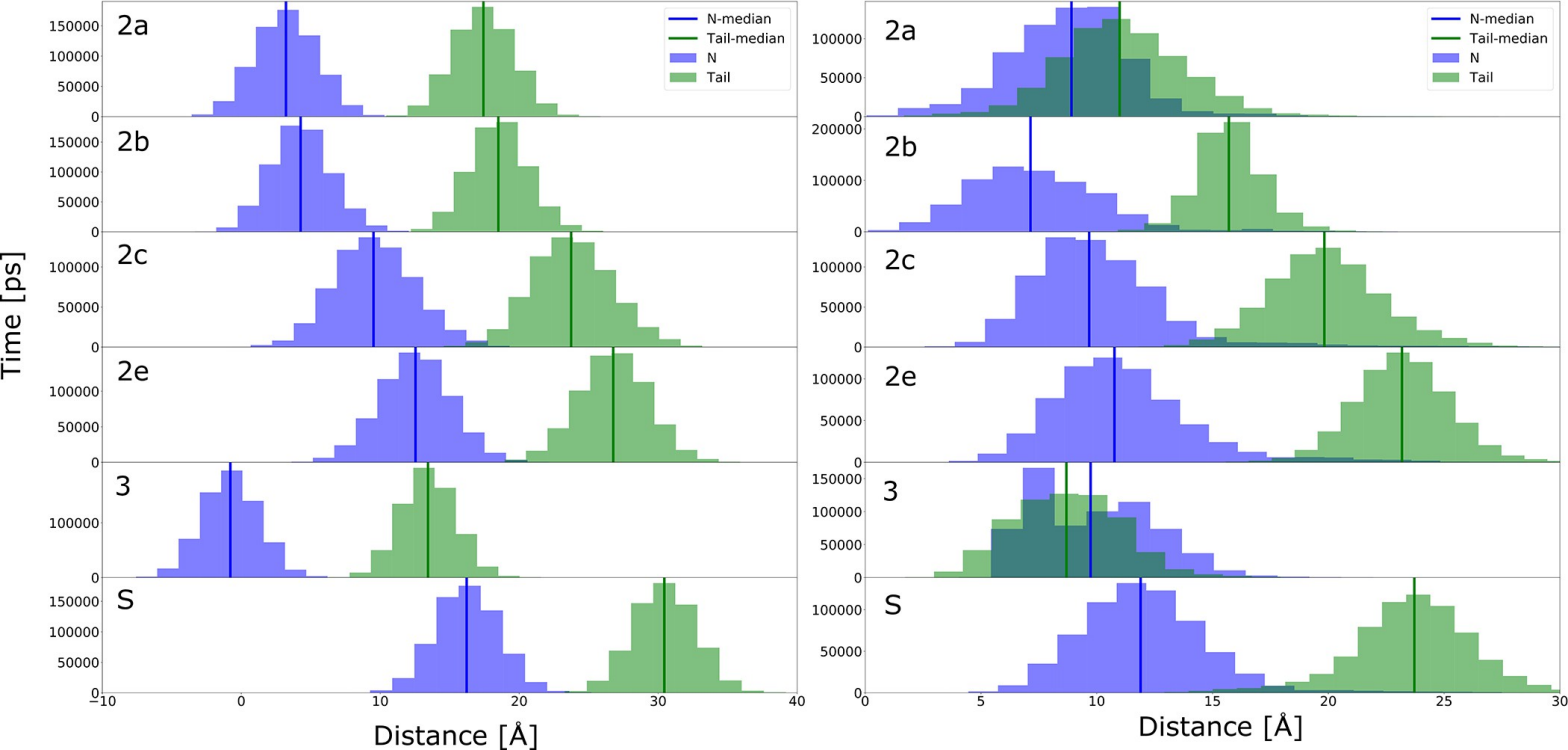
(left) Height distributions of each channel’s exit above the nitrogen (blue) and tail (green) layers of POPC in contact with the F’ and G’ helices. (right) The minimum radial distance distribution to the closest nitrogen (blue) and tail (green) for each channel’s exit.

The height above the nitrogen-plane and tail-plane of the membrane are plotted in Fig 5 (left column). The exit of channel 3 is centered right around the plane of the head groups. Channel 2a is above the head groups’ plane, while the exit of the S channel is the highest above the membrane, followed by 2e. These channels are more than 1.5 nm above the head group, therefore they open to solvent. Consequently, the detected channels allow ingress/egress to/from different locations within and above the membrane, but not from deep within the bilayer, as there is not one channel opening with a height centered around or close to a POPC tail. However, the F’ and G’ helices being embedded into the membrane perturb the bilayer, and thus the height above the membrane does not necessarily imply what environment the exit samples.

In Fig 5 (right column), the radial distances to the closest nitrogen and tail atoms are binned and plotted. Most channels show discretized distributions, which is anticipated based on the configuration of POPC; however, there is an overlap for channels 2a and 3. In these channels the exits sample the head and tail of the nearby lipids at the same distance; thus, these two channels almost equally open to the head group and tails of the membrane.

Taking into consideration the findings presented thus far, plausible correlations between ingress/egress preferred paths and nature of ligands can be drawn: (1) channel 2a is wide, with limited curvature, and bottleneck residues present at the exit of the channel, which points to either head or tail of the bilayer. This suggests hydrophobic product egress is most likely; (2) channel 2b is the widest, displays curvature, has medium length, and the highest throughput, with unrestricted to one locale bottleneck residues opening mostly to headgroups. Thus, besides being amenable to large ligands, channel 2b can function as either ingress or egress of mostly electrostatic molecules; (3) channels 2c and S both exit to the solvent. However, while channel S has bottlenecks which are not in a specific location, channel 2c’s bottleneck residues are the farthest from the heme; (4) channel 3 is the longest, with the most curvature and a mid-range bottleneck radius. Its bottleneck residues are far from the heme and it exits deeply into the membrane. Therefore, it is the most likely egress path for hydrophobic residues. Lastly, it is worth mentioning that the environment surrounding the channel exits is complex and variable and could impact ligand ingress/egress, as well as allosteric sites.

#### 3.2.1 Correlating channel positions with the environment

**Fig 6:**
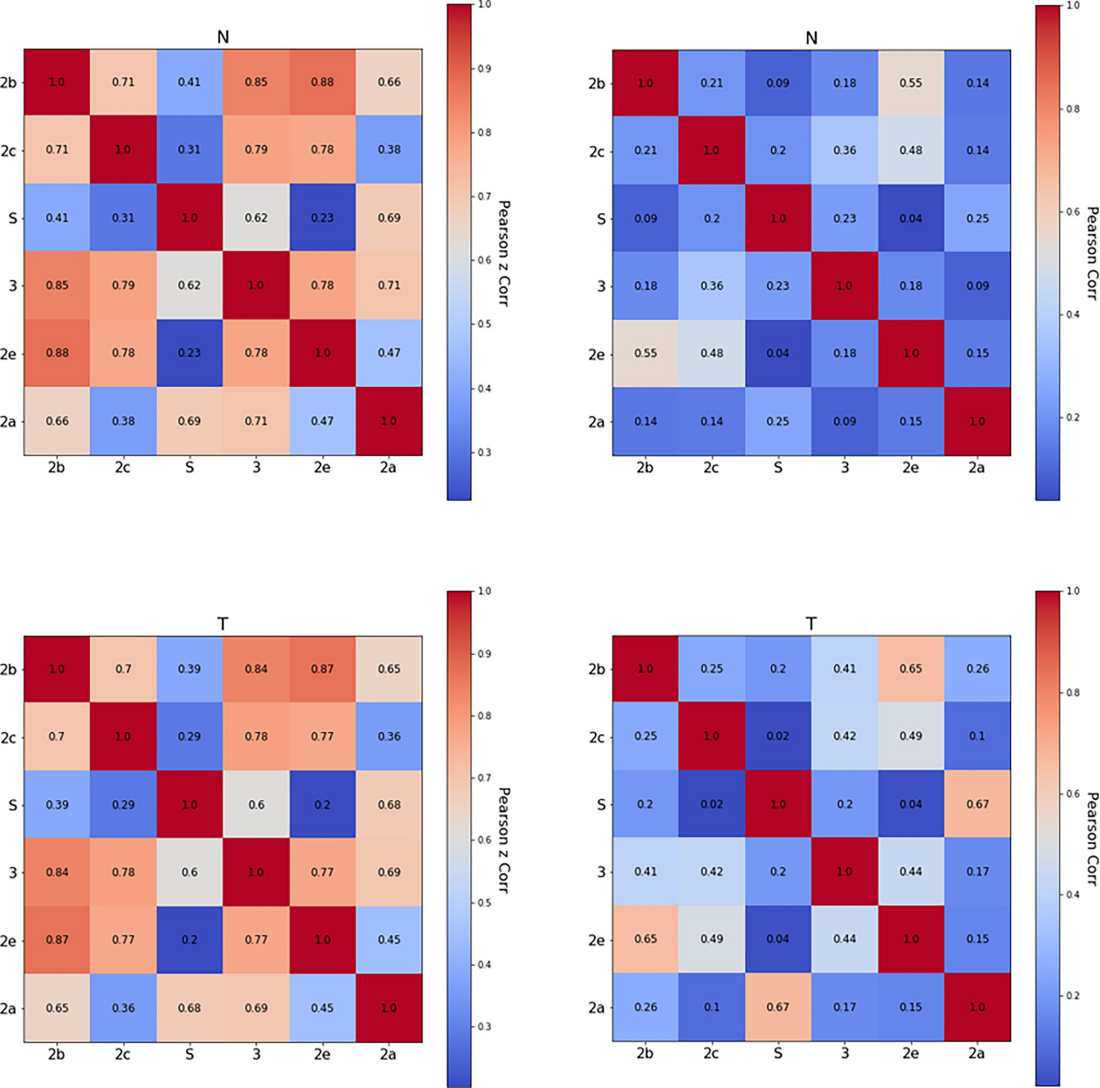
(left) Pearson correlation for the height above the nitrogen (top), tail-carbon (bottom) plane. (right) Pearson correlation for the radial distance to the closest nitrogen (top), tail-carbon (bottom) plane.

The Pearson correlations for the height above the nitrogen, phosphorous, and tail atoms for each channel and the respective radial distances are displayed in Fig 6, left and right panels, respectively. Correlations are overall high. The only low ones correspond to pairs S and 2e, and 2a and 2c. These channels are at almost opposite sides of the enzyme and correlation between them is lost. However, the correlation of the closest radial distances is high when channel exits face toward the same direction (2b-2e, 2c-2e) in reference to head groups. In contrast, some neighboring channels display high correlations with respect to the tails such as 2a-S, 2b-3, 2e-3. These stem from the displacement of the head groups by the enzyme, thus exposing the lipid tails to the macromolecule.

Earlier studies reported that the binding of the enzyme to the membrane does not impact protein flexibility significantly [50]. A perfectly rigid body would have perfect correlations for the height above each plane. High correlations between distant channel exits would suggest the macromolecule was somewhat rigid. Our results show no large-scale correlation between distant channel exits (S and 2e, 2a and 2c, S and 2c, 2a and 2e, 2b and S). This implies that a channel exit randomly changes height above the different planes in an uncorrelated manner with channel exits on the opposite side of the protein. Radial correlation suggests that a channel exit is sampling its lipid/cytosol environment independently of other exits.

### 3.3. Gating residues control access to and from the active site from the channels

Analysis of channels was performed on snapshots in which they were detected. However, it has been reported that CYP3A4 has gating residues (see Table 2) [50] which are typically in the catalytic site, and they may block egress of the ligand from the cavity. The following sections detail behavioral patterns of these gating residues.

#### 3.3.1 Distance distributions

**Fig 7:**
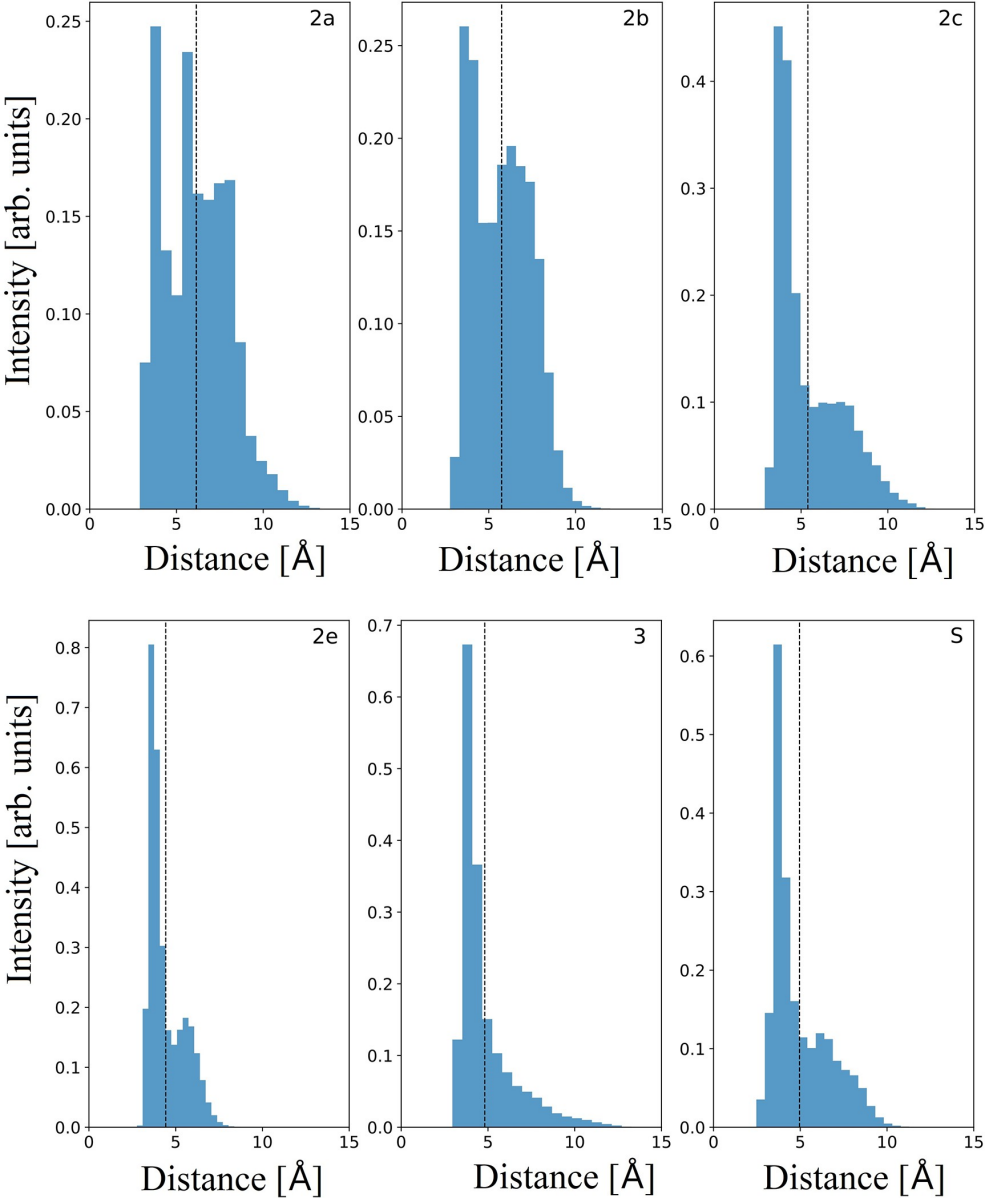
Distance distributions of each channel’s gating residues from all simulations for the membrane-embedded CYP 3A4. The dotted line represent the mean of the distribution.

The cumulative distance distributions of the gating residues were found to be at least bimodal in all pairs and are shown in Fig 7. Gating residue pairs have distinct peaks at around 0.4 nm, which is when they close the channel. This is particularly prevalent with the 2c, 2e, S, and 3 channels, where the distribution has very little population beyond about 0.5 nm. We consider this to be the closed state of apo CYP3A4. The remaining two channels are less likely to hamper the ingress of ligands, although they still spend a proportion of their time in the closed state (see Fig 7). Our findings are in qualitative agreement with Benkaidali et. al., [36] who reported the primary channels are 2f and 2b, corresponding to our 2a and 2b, respectively. Our MD simulations show that while the gating residues of 2a and 2b do close, they are significantly more likely to be open, when compared to gating residues of the other four channels. This is further evidence that 2a could be an egress channel since the gating residues would allow access from the active site and the bottleneck residues are only encountered near the head group. Similarly, 2b is likely an egress channel for the same reasons, although the bottlenecks may appear closer to the active site; however, it has a higher throughput. Moreover, our findings agree with Lonsdale et. al., [50] whose open/closedness averages are the same with ours. They reported a percentage of open gates at 40.8 ± 2.2% for the membrane-bound apo CYP3A4 state; our corresponding value for open gates (gates with distances larger than 0.48 nm) is 46.9 ± 21.9%. The difference lies mostly in the standard deviation. The discrepant standard deviation is attributed to a 100-fold increase in our simulation time; we ran 16 independent trajectories totaling 4400 ns, while they only had three runs amounting to 150ns total. Our longer simulations resulted in many more transitions than they observed, which also led us to identify more states.

#### 3.3.2 Inter-channel relations

**Fig 8:**
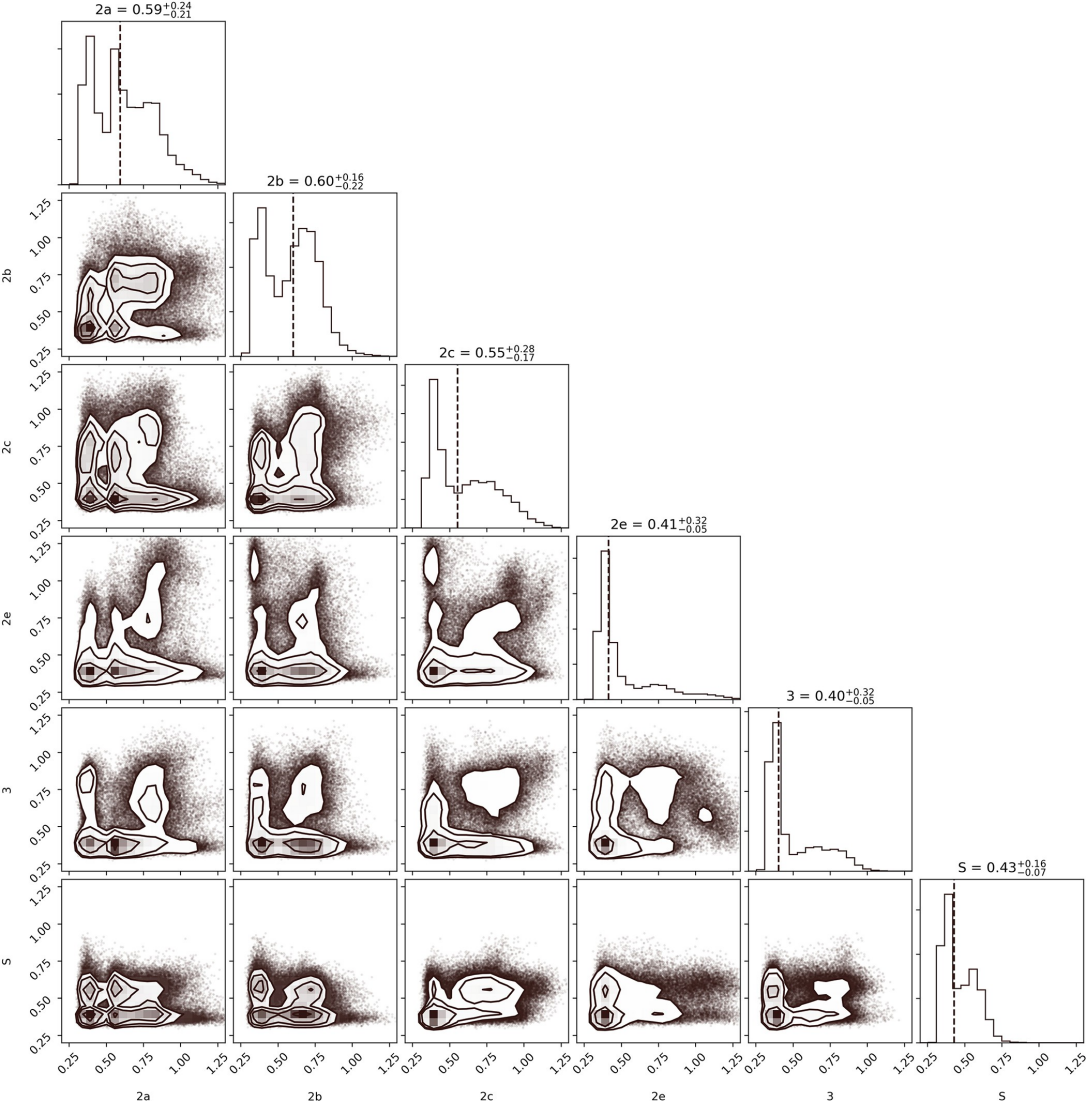
Dual distance distributions for each gating residue pair are plotted against each other gating residue pair. The total distance distribution is again (Fig 7) plotted at the top with the mean as dotted line and its value in the title, along with the 0.5 quintile for upper and lower errors. All values are in nanometers.

In an effort to explore whether an inter-gating residue correlation exists and how these residues behave in response to one another, distance distributions of each pair are plotted in Fig 8. The large population of states sampled in the bottom left corner shows that the gating residues remain closed in that both pairs maintain a small distance between their residues at the same time. The 2a and 2b residue pairs are sampled primarily parallel to the horizontal axis, at the bottom of each plot. Thus, they open and close almost independently of the other channels, but not with one another (Fig 8, 2a and 2b columns). In fact, the plot for the 2a-2b gates’ distribution has a significant population in the middle, a region where both gates would be open at the same time. The same observation can be made, even though to a lesser extent, for gates 2a-S and 2c-2e. We quantify these results using the Spearman Correlation measure and see that the values fluctuate significantly around the mean, resulting in standard deviations approximately equal to the means. We conclude the correlations are sporadic. The correlation in openings of gates 2c-2e is easily understood as both include F108. The other correlations are the result of residues in the gate sterically interacting with residues of another gate.

The correlations seen Fig 8 are without lag time. We also explored the possibility that these gates are correlated with some amount of lag time, by testing for all lag times in increments of 100 ps and averaging over all trajectories. The largest correlations were found with no lag time and sharp drops in the correlation were seen by 500 ps. Given that none of the gating residues is charged, nor do they form significant hydrogen bonds with each other, this finding is expected. However, it also suggests that backbone motions are not likely to drive any correlations. Thus, we suggest gateways open and close *randomly*, are not driven by larger motions, and correlate because the same residue is involved or the gating residues interact sterically.

#### 3.3.3 Transition probabilities of gating residues

**Fig 9:**
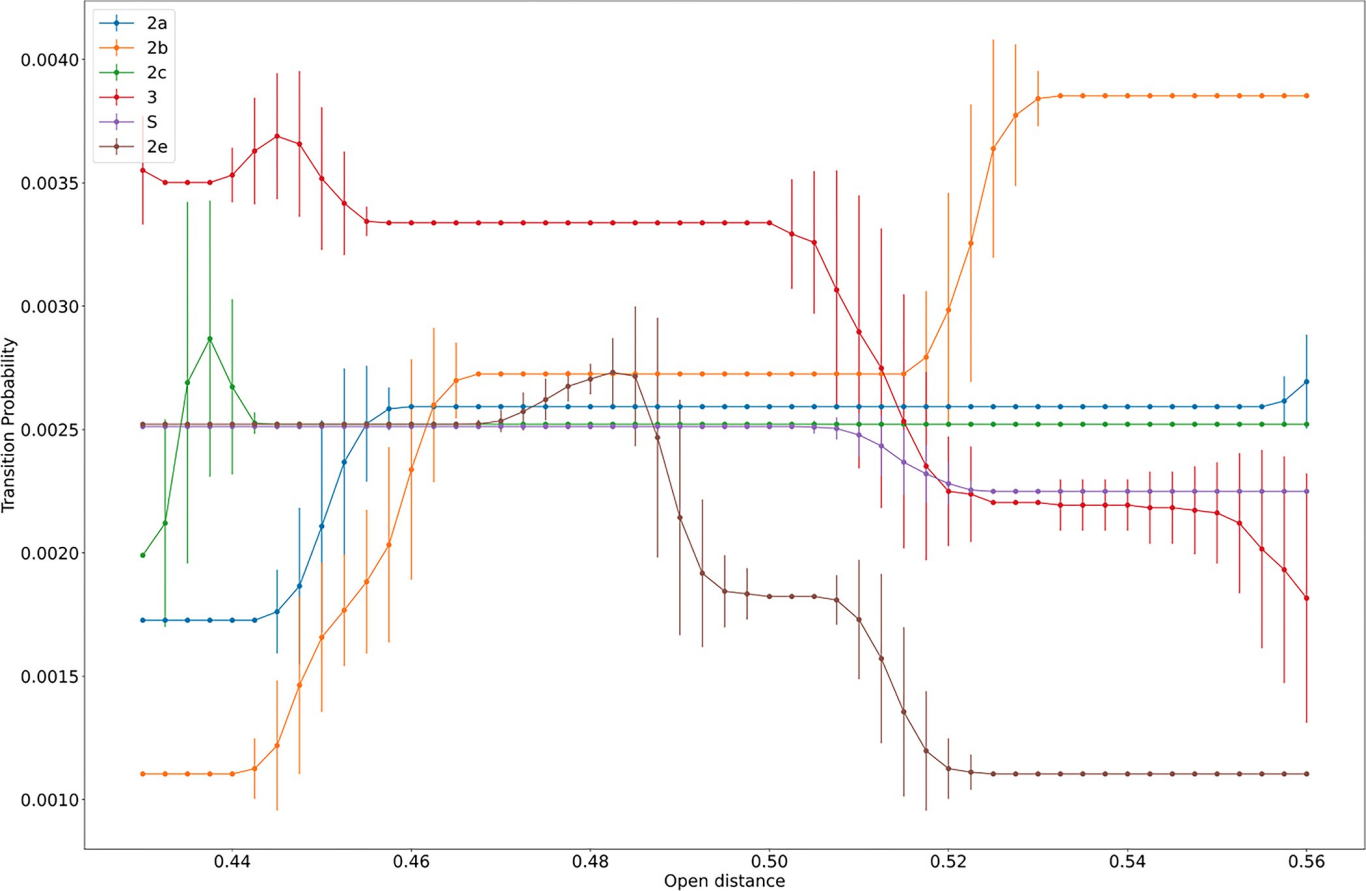
Transition probabilities for each gateway residue pair as a function of the distance chosen to differentiate openness from closedness.

Transition probabilities between open and closed states for the gateway residues depend on the chosen distance between them. Distance was varied from 0.43 nm to 0.56 nm. Probabilities for an open state to transition to any closed one (and vice versa, since transitions are symmetric), as a function of the chosen distance, are displayed in Fig 9. Regions where the transition probability is flat indicate that the dominant conformations for the gating residues in all Hidden Markov Model (HMM) states are different from the specified distance. Regions where the transition probability changes, and/or has a large standard deviation mean some of the dominant conformations in one or more of the HMM states are close to that distance.

The S-channel gives the simplest behavior as its transition probability remains constant to about 0.51 nm, when it starts to decrease and reach a lower plateau. Thus, gateway residues have the same probability of transitioning, regardless of the definition of open or closed from 0.43nm to 0.5 nm. This means the HMM states have dominant conformations, which are not close to that range, and the gating residues have stable conformations for those HMM states that differ significantly in their distances. Further support to this explanation is provided by the negligible standard deviations in all but the transition regions. Reaching a new plateau is indicative of changes pertaining to HMM states being open or closed. The value of the transition probability of about 0.0025 is in the middle of the range. Thus, the S-channel gateway assumes clear open or closed conformations for all assessed distances with an average transition probability.

The 2c channel behaves similarly to S, with two major differences. First, the probability fluctuates and has a large standard deviation with respect to small values of the boundary (0.43–0.45nm). Thus, the gateway separations for the HMM states are near these boundary values. Secondly, the transition probabilities remain constant for large boundaries. This means the sidechains (phenylalanines) have stable rotamers which allow for a clear open or closed designation for each HMM state, with slight variations as evidenced by the negligible standard deviations.

The other gateways have more complex variations with the boundary distance, suggesting some of the HMM states find conformations near the boundary distance which in turn result in large changes in the transition probabilities; this is attributed to designation changes of one or more HMM state(s) (open to close for instance). The most stable boundary distance is 0.5 nm and it remains stable throughout the 100 different models, as it has negligible standard deviations and is part of a plateau for all six gating residue pairs. In other words, all gating residue pairs have HMM states with stable conformations away from 0.5 nm. At this boundary value, channel 3 is the most likely to transition with almost double the probability of the lowest transition gateway, channel 2e. All others have roughly the same transition probabilities.

#### 3.3.4 HMM model

**Fig 10:**
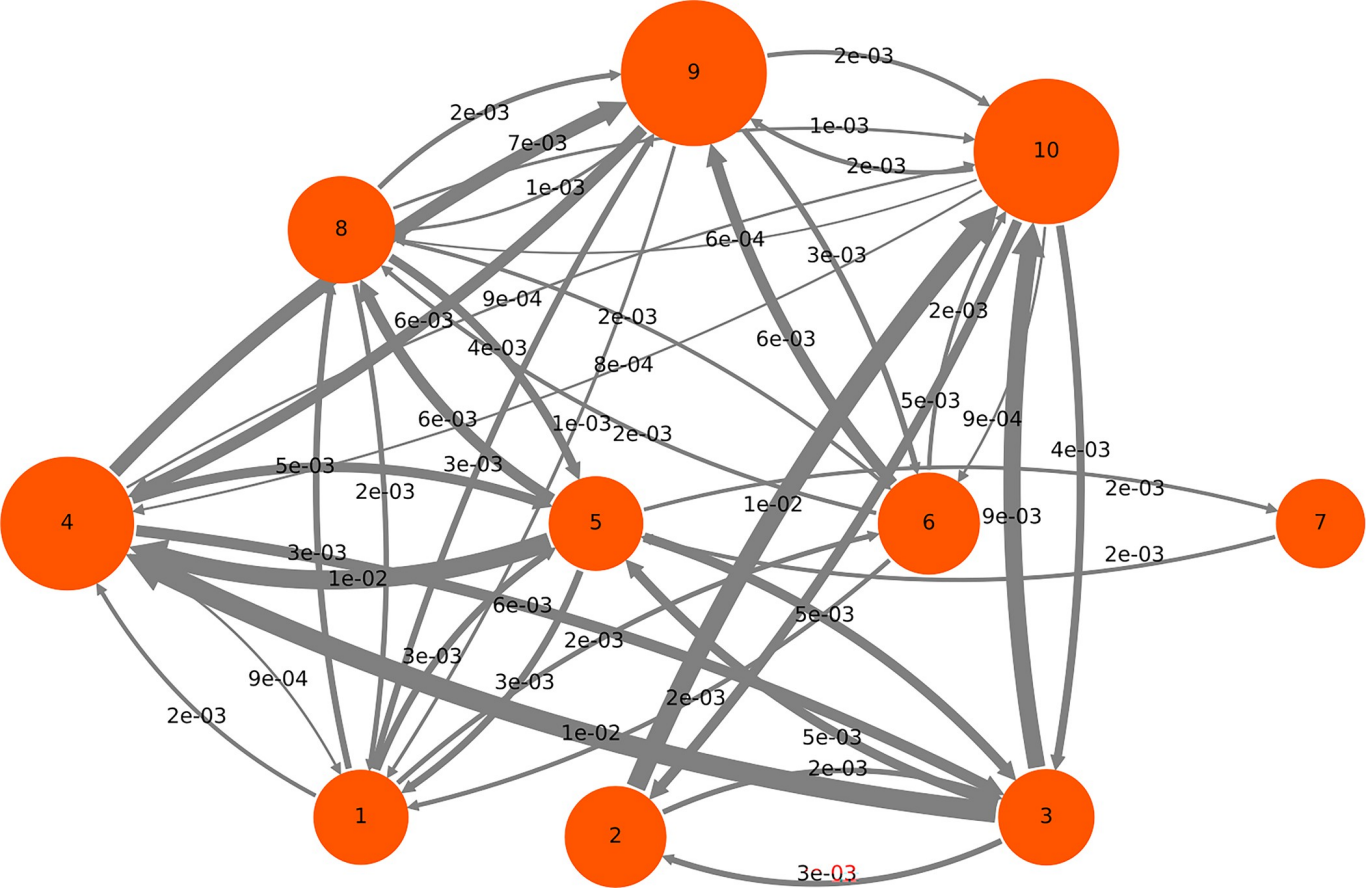
Ten-state Hidden Markov Model (HMM) of the apo CYP 3A4 with circles representing the state equilibrium population. Dominant transitions (with probabilities larger than 10^−5^) are depicted as arrows. Thickness is proportional to respective probability.

The 10-state HMM model found to represent the system most effectively is shown in Fig 10. States are depicted in circles proportional to their equilibrium population, while transition probabilities are listed if greater than 10^−5^. Equilibrium populations of each state are given in Table 4, while open or closed states are designated by O or C, respectively, based on a boundary distance of 0.5 nm. State 9 is almost three times more populated than the lowest populated state 7, and it has all channels closed. Conversely the least populated state 7 has all the gateways open except for 2e. Direct transitions between these states were not observed (see transition matrix in S1 File).

**Table 4:** Equilibrium populations of each state as a function of boundary distance.

	Channel						
HMM state	2a	2b	2c	2e	3	S	Equilibrium Population
1	C^a^	O	O	C	O	C	0.071
2	O	O	C	C	O	O	0.081
3	O	O	C	C	C	C	0.072
4	O	O	C	C	C	C	0.140
5	O	O	O	C	O	C	0.070
6	C	C	O	O	C	O	0.081
7	O	O	O	C	O	O	0.062
8	O	C	O	O	C	C	0.090
9	C	C	C	C	C	C	0.167
10	O	O	C	C	C	O	0.166
Open probability	0.681	0.662	0.374	0.171	0.284	0.390	

^a^Open states are denoted by ‘O’ and closed by ‘C’.

The largest transition out of the most populated state 9 is to state 4 (3^rd^ most populated) and it results in the opening (distance greater than 0.5 nm) of the gateways for 2a and 2b channels. Moreover, state 3 has an identical set of open and closed states to those of state 4 main egress channel, as it is rarely blocked by gating residues, has a high throughput and is wide despite its bottlenecks.

The 2c and S gating residues are the next tier of possible egress channels. These are significantly more likely to be closed and are both long with low throughputs. They both exit to the solvent, but 2c has its bottlenecks near the exit of the channel, while S tends to have its bottlenecks near the catalytic site. Thus, 2c represents a more likely egress route for hydrophilic metabolites than S, in spite of its larger average bottleneck size. Channel 3 is the longest, with a high curvature, and is frequently blocked by the gating residues. Its bottlenecks tend to be far from the catalytic site. Thus, it is an unlikely egress path for many metabolites, even though it exits deeply into the membrane. Lastly, channel 2e is almost always blocked by the gating residues; it would be the least likely to function as an egress channel even though its bottlenecks are near its middle and it is straight.

## 4. Conclusions

We present our findings from multiple MD simulations employing CAVER to quantify the channels, and HMM to characterize the behavior of the gating residues for the cytochrome P450 3A4. Channel throughput and curvature are dependent upon the size of the ligands, as they are moving away from the heme, with 2b having the largest throughput and 2c the lowest. When considering bottleneck residues, channels 2a and 2c display their residues the farthest away from the heme, thus modulating drug ingress the most. We also discuss the location of the channels relative to membrane, and suggest their potential for ingress or egress, finding that 2a is most likely an egress channel for hydrophobic products, whereas 2b is a putative ingress and egress channel for electrostatic ligands. Channel 2c and to a lesser extent the S channel are likely egress for hydrophilic products. Finally, gating residues are investigated, concluding that 2a and 2b are more likely to be open, when compared to other channels; they also represent the dominant egress channels.

Overall, we find that the channels do not display coordinated motion, openings, or location with respect to the membrane. They randomly transition between different conformations. Gateway residues also behave in a random fashion, but seemingly indicate which channels are likely to be favored for egress (2a and 2b), as most of the time all their gateways are closed. In conclusion, understanding the equilibrium behavior of the gating residues and channels for the apo state provides a foundation for further elucidation of allostery and mechanistic details following ligand binding using HMM’s.

## Supporting information

S1 File (DOCX)
