# Supplementary material for: Equilibrium landscape of ingress/egress channels and gating residues of the Cytochrome P450 3A4
Source: PLoS One. 2024 Mar 18;19(3):e0298424. doi: 10.1371/journal.pone.0298424 (PMC10947690; doi:10.1371/journal.pone.0298424)

**Supplementary Information**

The decision of how many states to use to create a hidden Markov Model (HMM) depends on the system of interest. Below we plot the fractional uncertainty (Eq. 1) for each HMM model. For every number of states (from 6 to 20) we created 100 independent HMMs. Thus, from 10 to 11 in the plot below are the 100 dots representing the average fractional uncertainty for each 10-state HMM model created.


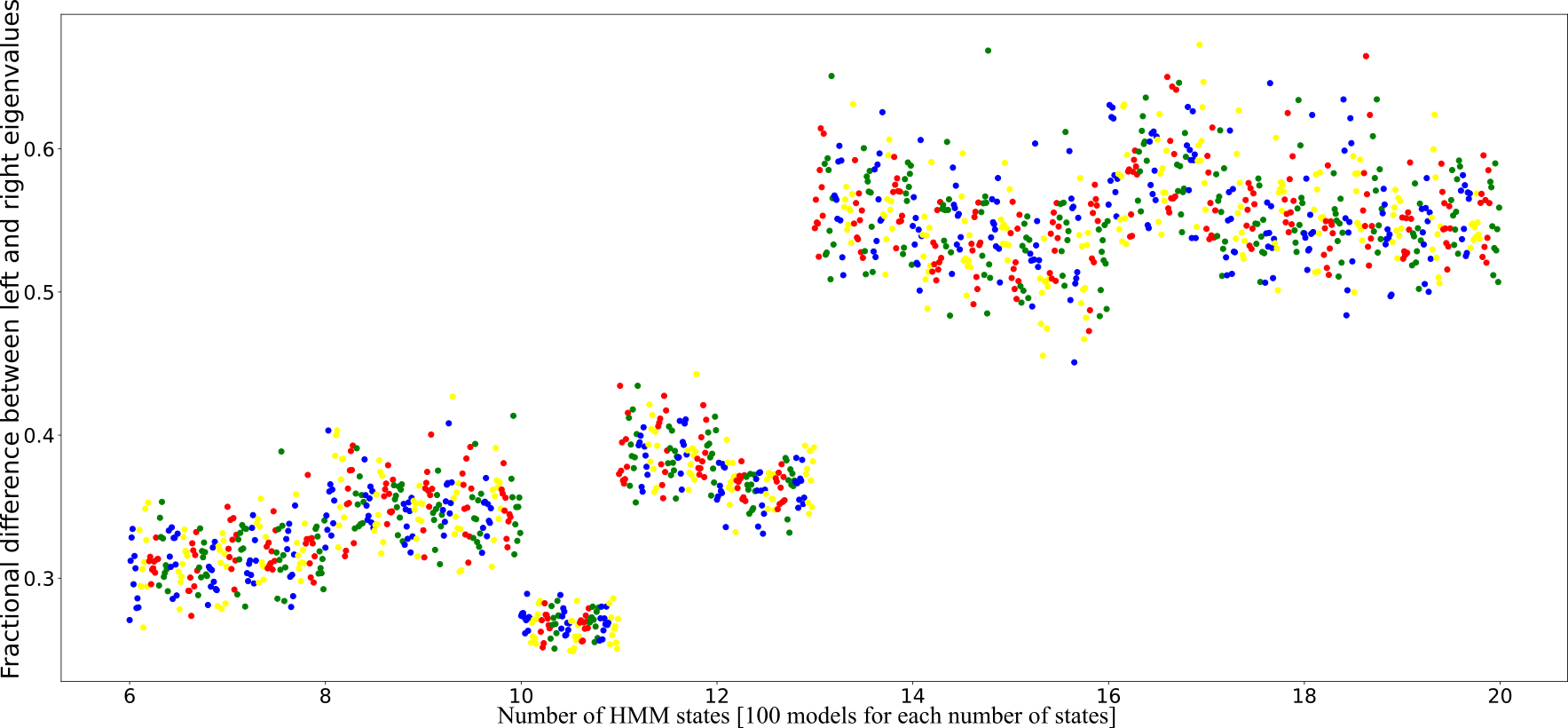


A clear minimum is seen from 10-11. The model with 10-states which had the absolute lowest fractional uncertainty (located at 10.48) was chosen for the detailed analysis of section 3.3.4. The transition matrix of this 10-state HMM model is given in the table below.

| Transition Matrix | | | | | | | | | | |
| --- | --- | --- | --- | --- | --- | --- | --- | --- | --- | --- |
| HMM State | 1 | 2 | 3 | 4 | 5 | 6 | 7 | 8 | 9 | 10 |
| 1 | 9.85E-01 | 3.58E-05 | 1.93E-95 | 1.73E-03 | 3.39E-03 | 2.23E-03 | 1.05E-03 | 3.01E-03 | 3.10E-03 | 1.33E-04 |
| 2 | 3.12E-05 | 9.87E-01 | 2.45E-03 | 1.09E-04 | 3.66E-05 | 3.80E-04 | 1.63E-04 | 1.50E-173 | 1.33E-102 | 1.02E-02 |
| 3 | 1.88E-95 | 2.73E-03 | 9.73E-01 | 1.09E-02 | 4.63E-03 | 3.67E-146 | 1.63E-05 | 1.55E-05 | 1.42E-20 | 8.91E-03 |
| 4 | 8.71E-04 | 6.29E-05 | 5.63E-03 | 9.80E-01 | 4.89E-03 | 3.48E-04 | 8.37E-55 | 2.16E-04 | 7.22E-03 | 9.13E-04 |
| 5 | 3.42E-03 | 4.24E-05 | 4.80E-03 | 9.82E-03 | 9.74E-01 | 1.20E-04 | 1.55E-03 | 5.53E-03 | 1.74E-04 | 8.27E-04 |
| 6 | 1.93E-03 | 3.78E-04 | 3.27E-146 | 5.99E-04 | 1.03E-04 | 9.88E-01 | 1.09E-04 | 1.77E-03 | 5.70E-03 | 1.79E-03 |
| 7 | 1.19E-03 | 2.12E-04 | 1.90E-05 | 1.89E-54 | 1.74E-03 | 1.43E-04 | 9.96E-01 | 4.39E-04 | 8.57E-210 | 5.97E-04 |
| 8 | 2.36E-03 | 1.35E-173 | 1.25E-05 | 3.37E-04 | 4.28E-03 | 1.60E-03 | 3.03E-04 | 9.88E-01 | 2.23E-03 | 1.15E-03 |
| 9 | 1.31E-03 | 6.45E-103 | 6.14E-21 | 6.06E-03 | 7.27E-05 | 2.78E-03 | 3.19E-210 | 1.20E-03 | 9.87E-01 | 1.94E-03 |
| 10 | 5.67E-05 | 4.96E-03 | 3.89E-03 | 7.71E-04 | 3.48E-04 | 8.76E-04 | 2.23E-04 | 6.24E-04 | 1.95E-03 | 9.86E-01 |

**Residue Occurrence in the 2e Channel**
The percentage occurrence for each residue to be included in the bottleneck trio is shown on the plot below.


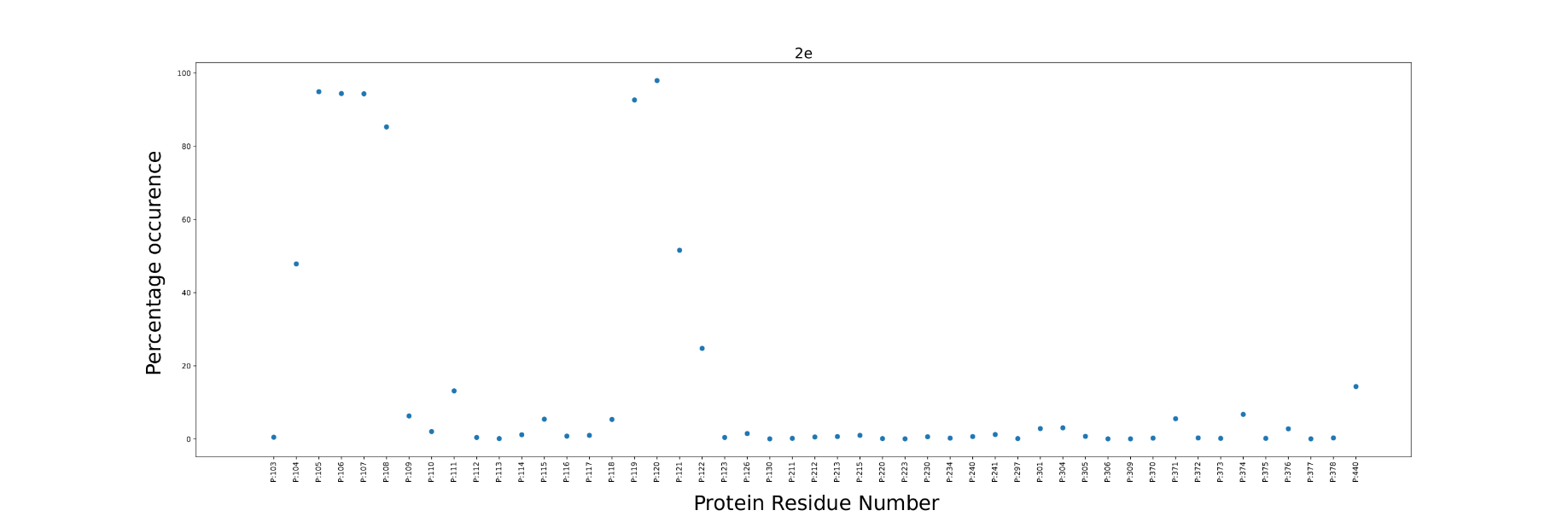

Supplement: S1 File — (DOCX) [file pone.0298424.s001.docx]
